# FACTORS THAT INFLUENCE PSYCHOTROPIC MEDICATION USE

**DOI:** 10.1093/geroni/igz038.2062

**Published:** 2019-11-08

**Authors:** Barbara Resnick, Elizabeth Galik

**Affiliations:** 1 University of Maryland School of Nursing, Baltimore, Maryland, United States; 2 University of Maryland, Baltimore, Maryland, United States

## Abstract

Understanding the factors that influence psychotropic can guide reduction in use of these medications. This study described predictors of psychotropics use among residents with moderate to severe cognitive impairment. This was a secondary data analysis using baseline data from the first 341 residents in the EIT-4-BPSD trial. Predictive measures included demographics, agitation, resistiveness to care, depression, cognition, pain, facility factors and state. Overall 63% (n=211) received at least one psychotropic medication, 16% (n=52) an anti-seizure medication, 23% (n=77) an anxiolytic, 30% (n=99) an antidepressant, 2% (n=8) a sedative hypnotic, 28% (n=93) an antipsychotic medication, and 9% (n=29) an opioid. Model testing explained 9 to 15% of psychotropic medication use. There were high rates of psychotropic medication use and a limited association between demographic factors, behavioral symptoms, and psychotropic medication use. Continued research is needed to explore additional factors associated with psychotropic medication use such as beliefs of providers.

